# Nursing Students Explore Meaningful Activities for Nursing Home Residents: Enlivening the Residents by Cultivating Their Spark of Life

**DOI:** 10.3390/nursrep11020022

**Published:** 2021-04-01

**Authors:** Britt Øvrebø Haugland, Tove Giske

**Affiliations:** Faculty of Health Studies, VID Specialized University, Ulriksdal 10, 5009 Bergen, Norway; tove.giske@vid.no

**Keywords:** nurse-patient interaction, qualitative research, person-centered care, meaningful activities

## Abstract

International research focuses on person-centered care, quality of life, and quality of care for people living in long-term care facilities, and that it can be challenging to improve the quality of life for residents with dementia. The aim of this study was to explore ways of developing appropriate person-centered activities for nursing home residents based on what would be meaningful for them. A qualitative explorative design was chosen. Twelve students each year over a three-year period participated in the study (altogether 36). Each student tailored joyful and meaningful activities for two nursing home residents and wrote eight reflection journals each (altogether 284). Additional data came from eight focus group interviews with the students. Data were analyzed using qualitative content analysis. The main theme was “Enlivening the residents by cultivating their spark of life”. Two main categories were identified: (1) “Journeying to meaningful and enlivening (enjoyable) activities”, and (2) “Expressions of enlivening”, It is possible to tailor meaningful and enlivening activities together with the individual person with dementia. Involvement and engagement are necessary to understand the verbal and nonverbal expressions and communicate with the individual resident.

## 1. Introduction

There has been a growing international interest in the quality of life and the quality of care for residents living in long-term care or nursing homes [1,2,3,4]. The principle of local care, introduced by the World Health Organization (WHO) [5], builds on evidence that most people want to live in their home or familiar surroundings for as long as possible. For various reasons, such as severe dementia, some old people are unable to live at home. Dementia affects a growing part of the global population. The WHO estimates that, currently, dementia affects approximately 50 million people. The number is projected to grow to 82 million by 2030 and 152 million by 2050 [6]. Providing appropriate care for people in the late stages of dementia often involves transfer to long-term residential care. In Norwegian nursing homes, about 80% of the residents have dementia [7,8], with a significant portion of the persons lacking the ability to communicate and thus the ability to express their needs, including what is important and meaningful to them. To be meaningful, the activities have to be person-centered and tailored to the individual resident [9,10]. Individual preferences and abilities have to be assessed and addressed, which also increases the everyday interaction between the caretaker and the nursing home resident [11,12]. To understand the individual person, you must know something about this person’s past. When a person achieves continuity with their personal past, it can aid the person in feeling a sense of coherence across their lifespan [13]. The importance of the continuity of self is also mentioned by O’Sullivan [14].

In the literature, the quality of care in nursing homes is measured by several parameters, such as autonomy, social relationships, joy of life, and meaningful activities [1,3,15,16,17]. Studies have suggested that the relational qualities of the nurse-patient interaction are key to the residents’ sense of self-worth and well-being [15,16]. Several studies show that nursing home residents (and their families) are, to a certain extent, satisfied with the physical care but lack meaningful days, social contact, and the ability to make their own choices [18,19]. When considering the quality of care in nursing homes, it is crucial to assess all aspects of life that affect health such as the physical, social, mental, and spiritual aspects to understand the person as a complete human being. Studies have shown the importance of meaningful activities and stimulation of the overall health for an individual [1,19,20,21,22,23].

Morgan-Brown et al. [18] studied the social and occupational engagement of the nursing staff and the occupational coordinators in two nursing homes for persons with dementia in Ireland. Only 10 % of the time of the staff was spent on interactive activities with the residents. The rest of their time was task-oriented and routine-based, where staff were doing things to and for the residents. Due to lack of interaction, the residents were sleeping in their chairs or staring into space for most of the day [18]. When residents with dementia spend most of their time having little sensory stimulation, rarely conversations, or limited engagement in meaningful activities, this could lead to undesirable behavioral and psychological symptoms of sensory deprivation, and/or social isolation [24]. Edwards et al. [25] indicated that a nursing practice based on lack of interaction and invitation for participation reinforces the residents’ dependence and institutionalization.

As the nursing home is actually the home of the residents during the last years of their life, the sense of home is important and is influenced by a set of qualitative elements identified by van Hoof et al. [26] and Røen et al. [27]. The qualitative elements include the building and the interior design, eating, drinking, autonomy and control, involvement of relatives, engagements with others, quality of care, and connection with nature and the outdoors. The connection with nature and access to outdoors activities is of importance to provide a sense of home for nursing home residents [28]. O’Sullivan [14] presented a protocol for a leisure-activity program expected to enhance health, quality of life, and identity of persons living in long-term care facilities. She described five key principles for what encourages people to participate in activities: the activities must be experienced as meaningful, challenging, a continuation of the self, need-based, and giving a sense of community. Ouyang et al. [19] showed that leisure activities have a positive effect on the health, acting as a buffer on age-related stress of institutionalized old persons. Brooker, Woolley, and Lee [12] developed the Enriched Opportunities Program, meant to ensure that the residents reach their potential for well-being. Central to the program were the senior staff members, who in this program were named “Locksmiths”. The Locksmiths work with the staff members and the residents to ensure that they reach their potential by assessing and developing interventions tailored to the individual resident with dementia.

Nationally and internationally, in governmental plans and research, there has been a focus on the term “person-centered care” (PCC) for persons living with dementia [6,7,29]. The WHO uses the term PCC to empower people to take charge of their own health rather than being passive recipients of services. Health services should empower people with dementia to make informed choices and decisions about their care [6,29]. PCC requires healthcare services and staff to treat the residents with dementia in a positive manner, including respect, dignity, and promoting autonomy. To support the philosophy of PCC, Brooker and Latham [30] developed a framework called VIPS. The VIPS framework consists of four key elements: (1) Valuing the service user and service staff; (2) Individualized care—that is, treating people as individuals; (3) Personal perspective, or looking at the world from the perspective of persons with dementia; and (4) Social environment, or the entire human-relationship environment, including the staff and service-user relationships. Internationally, the key elements have been used as guiding principles to promote PCC for people living in long-term care facilities [31,32,33], and there has been an increasing amount of research on PCC at the organizational level, in terms of education, and job satisfaction [27,31,34,35,36].

However, there is little research on how to unpack and tailor meaningful activities to the individual nursing home residents, the majority of whom suffer from different degrees of dementia. The aim of this study is to explore different ways of communicating with nursing home residents. We want to explore ways to develop appropriate person-centered activities for nursing home residents based on what the individual residents would experience as meaningful and fulfilling. This article presents the findings from a three-year mandatory program by first-year nursing students in their 8 weeks nursing home placement.

## 2. Method

### 2.1. Study Design

This study deals with a relational phenomenon, where communication and relations between the carers and the residents with different degrees of cognitive impairments are at the core. We therefore chose a qualitative explorative design as the best design and we used qualitative content analysis [37,38].

### 2.2. Participants and Context

First-year undergraduate nursing students who were ready for their first eight weeks of clinical practice in a nursing home were invited to participate in the project. Twelve out of a cohort of 95 students participated each year over a three-year period; in total, 36 students participated in our study. Each of the students agreed to follow two primary residents during their eight weeks of placement. The students were encouraged to involve themselves with the individual resident and they wrote reflection journals every week about their interactions with the residents. In the reflection notes they described the planning, implementation, and own reflections after the completed activity with the resident. According to the principle of local care [5], people are not admitted to the nursing home until they are unable to live in their home anymore; consequently, most inhabitants in the nursing homes in Norway are very frail, and about 80% have dementia [8]. Our study took place in an urban-area nursing home with about 100 residents.

### 2.3. Data Collection

We collected data from the 284 reflective journals written by the 36 students who consented to take part in the study. For the reflective journals, the students were assigned to write about the planning and implementation of the tailored activities, the residents’ verbal and nonverbal expressions and reactions, and their own reflections. In addition, we also collected data from eight focus group interviews with the same students. The first author, the teacher of the students, and a key collaborator from the nursing home moderated the focus group interviews. Focus group interviews provide a forum for participants to share and discuss personal experiences and to bring forth their diverse views and experiences [39]. In the focus group interviews, the students were invited to talk about the activities and reflect on their experiences regarding the residents’ expressions in relation to the personalized activities and social contact. Each interview lasted for about 60 min and was digitally recorded and transcribed verbatim.

### 2.4. Data Analysis

The reflective journals were read consecutively as the first author received them over the three-year period and thus complemented the focus group interviews. The data from the students’ reflection journals and focus group interviews were analyzed using qualitative content analysis [37,38]. The first author read the reflective notes, and both authors read the transcribed focus group interviews individually and coded them manually in the margin of the transcript, for diversity of meanings. We met to share the codes and discuss which codes belonged together and how the transcribed focus group interviews corresponded with the reflection journals. Initially, we developed three categories. The three categories were variations in patients’ conditions, tailored activities, and expressions of enlivening. We went back to the data to make sure we had identified all the nuances for each category and to check whether the categories were externally heterogeneous. We continued analyzing by reading through the material again, and gradually we condensed our data and grouped them into two categories to make them distinctly different from each other [37]. Our analysis of the data from the last group of students confirmed the categories we had developed, and thus our data were saturated. Along the way, we discussed what the latent meaning of this study could be, and we tried different ways of expressing the main theme of this study.

### 2.5. Ethical Considerations

The Norwegian Social Science Data Service (NSD #25963 and #29852) approved the project under the condition that we obtain written consent from the students based on written and oral information. The transcripts of the focus group interviews and the reflection notes were anonymized. Student statements in the focus group interviews were referred to using pseudonyms. The reflective journals were each assigned a number as they were handed in, from 001 to 284.

## 3. Results

The main theme of this study was ‘Enlivening the residents by cultivating their spark of life’, which was consistent throughout our data. When a resident felt enlivened, they expressed it both verbally and non-verbally, and the attentive student could be guided by these expressions. What led to a spark of life was individual and had to be searched for with each nursing home resident based on their personality and cognitive and/or physical functioning. When analyzing our data, we found two main categories showing how the nursing home residents became enlivened by sensing the spark of life. The first category was ‘journeying to meaningful and enlivening (enjoyable) activities’, describing how the students and residents travelled together to search for and adjust to what was meaningful for each resident as the students interpreted the residents’ verbal and nonverbal expressions. The second category, ‘expressions of enlivening’, we carried out an in-depth analysis of how the students recognized and interpreted the expressions of enlivening among the residents. In real life, these two categories are intertwined.

### 3.1. Journeying to Meaningful and Enlivening Activities

The residents’ health conditions ranged from good physical strength and mobility to physical impairment, and some were even bedridden. The conditions also varied from good cognitive function to severe cognitive impairment, and the residents’ senses, such as vision and hearing, differed. Although most of the residents were diagnosed with dementia, they acted and functioned very differently:

“There is one that does not have language and has dementia, and another who has dementia and understands language but can give little and unclear answers. There is still another who is very quick, having both good language and good hearing.”

(Emma)

In order to facilitate activities that the individual nursing home resident found meaningful and enlivening, the students had to spend time and build a relationship with the resident. In that way they could become attuned and acquainted with the person and find out the interests of the individual resident. The students also had to search for ways to overcome the physical, cognitive, and communicative obstacles and tailor the time, space, and activities to the individual person. The students assessed which activities provided meaning and pleasure to the individual residents. To do so, the students asked the residents themselves, their next of kin, and the nursing home staff and read the resident’s records. However, they also found that they had to try, fail, and try again to find out what was joyful and enlivening for each resident. The students’ journeys of trials and failures while looking for enlivening moments were guided by conscious and careful observations of the residents. Students sought for activities and objects that could connect with the long-term memories and core persons in the lives of the resident. When the residents had difficulties expressing what they liked and disliked, the students were guided by what they saw as creating enthusiasm in the residents. For example, they looked at the photos on the wall in the residents’ rooms and looked at the residents’ photo albums together. As they viewed the albums, the residents would become very enthusiastic about specific photos, which provided the students with an excellent opportunity to start a conversation with the residents that could talk and thus to search for areas of interest that could induce sparks of life in the residents. The residents who still possessed the oral language skills showed enthusiasm and began to talk about the people or events shown in the pictures. When the residents had language impairment, the students tried to interpret what they saw in the photos or in the other objects that the residents had in their room. One student said the following:

“When the resident did not possess the language, we just had to take the time to find keys so they would open up more; we began to talk about many different things. Then, I looked at the expression of one resident, suddenly I said a word, and there I found a key. Then the resident just lit up, and there was the key.”

(Isabella)

In the reminiscence room, many of the residents disclosed interests in activities that were meaningful to them. While browsing a scrapbook with photos of various activities or showing different objects, some residents lit up and revealed their individual interests, a discovery the student could explore further. For example, one resident saw somebody knitting and told that she used to love knitting and had even run a knitting store. Another resident disclosed that she did not like knitting at all. In a quiz session later, it turned out that she knew the lyrics of all the children’s songs. This led to a singing session, following up on this resident’s passion for song and music.

Students could find help in the residents’ records; for example, one resident’s former occupation was noted to have been that of a shoe dealer. The student brought a child’s shoe to him, and they talked about shoes for a long time. The student even tested the resident’s sense of humor: “Have you fooled any customers? Yes, many, he replied” (Veronica). Both had a good laugh. Another resident used to be an architect and had designed several of the houses in the town. While being out on a tour in areas where he had planned the houses, he woke up and began to tell about his former occupation. The lack of personal information in the nursing documentation encouraged the students to assess and document the residents’ history, likes, and dislikes. By doing this, the students gave the person identity, and the resident became a unique person with a history. One of the students said the following:

“When we collected information and so on, we gave the person an identity as well. There was not just a resident who had a diagnosis but a person who still likes different things, and when they lose the ability to express it, it is very important to look for it.... I would like to, if I once lost the ability to express what I wanted, still have someone who was looking for it. To make it possible for me. To find out what I liked to do.... So it would be maintained.”

(Olivia)

Some residents were walking restlessly in the nursing home corridors and wanted to go out. These residents were very happy if someone could help them by facilitating “an outing.” Other residents felt ambivalent and initially did not want to try new things, such as leaving the ward or the nursing home, as they felt safe there. Some residents showed resistance to doing the things they had previously enjoyed, something that the students interpreted as an uncertainty and anxiety with respect to the unknown. One student reflected as follows:

“She really enjoys getting out of the ward but refuses to do so because of restlessness and anxiety. If a person in which she is confident can be with her, this becomes a very nice experience for her and enriches the rest of her day.”

(112)

Many of the residents had not been out in fresh air for many years due to mobility impairments. They were dependent on being transported in wheelchairs, which could be difficult in the middle of the city with busy streets and high curbs. Another obstacle was the harsh Norwegian winter weather with wind, snow, or slush and all the hassle of finding appropriate clothes and equipment. Other residents did not even have outer clothes for cold weather and needed to be wrapped in blankets to go out. Some residents were very quiet, did not ask for anything, and took little initiative but were very happy when they were offered to participate in activities. For residents who did not want to or were too weak to participate in outdoor activities or other more strenuous physical activities, students offered hand care, massages, and nail polishing. Students found such small gestures to be very rewarding for the residents as the residents showed confidence, became relaxed, and opened for conversation. It also boosted the residents’ self-confidence.

### 3.2. Expressions of Enlivening

The residents exhibited both verbal and non-verbal expressions that students interpreted as signs of enlivening in relation to individualized activities conducted together with the students. The residents with impaired or absent language function expressed themselves using particularly the mouth and eyes. One student said the following:

“It was particularly the smile, or what they did with their mouth or eyes. They could, for example, close their eyes and relax. Often, they opened their eyes more and focused on what happened. The corners of their mouth went up more, into a smile, showing their teeth, and dimples. Yes, it was the smile and the eyes.”

(Nora)

The body language also contained expressions of enlivening. The persons who usually had a closed posture could often pull their shoulders back, lift their eyes, and become aware of what happened around them. One student reported the following:

“She did not speak very much, but I noticed as we were singing, something she liked very much, she leaned toward me, took my hand, her eyes lit up. She held my hand, as if we were close friends, singing together. I think she appreciated it; it was just as if her eyes opened more and lit up. Then she smiled and sang at the same time.”

(Mia)

Many residents enjoyed getting out into the fresh air, being in touch with nature, and looking at trees, green leaves, and flowers. One woman was reminded of how she loved skiing as a young girl while sensing the fresh air on her face and observing snow on a mountaintop. These memories initiated a conversation with the student, who asked about the resident’s interests and what she used to do when she was younger. Another student read in a resident’s nursing journal that she used to love nature. The student took her out in a wheelchair, and although the woman had a serious language impairment, when she came out, she raised her head, looked around, listened to the birds, and started to make bird sounds. She seemed to enjoy it very much. The student said the following:

“In the evening, while her next of kin visited her, she was waving with her hands and pointed out the window. Her daughter asked if she had been outside, saying that she had pointed out the window and was so content. I think she managed to remember and was able to convey, even if it was difficult for her to communicate anything at all. Her family understood.”

(Isabella)

Students also reported that when they were out with residents for a short time in rough weather with snow, wind, and slush, they woke up.

“I first placed the wheelchair with her face toward the sun, when she said it was too bright, we turned the wheelchair a little. The resident expressed that it was delightful with the fresh air. She commented on the weather and the snow. We sat outside talking, and after 15 min, she said it started to get too cold and wanted to move inside again. The resident said it had been a nice little trip, and you could tell from her body language as well. She smiled and relaxed in her body and face all the time we were outside.”

(109)

As the residents were taken out of the nursing home, into other environments and surroundings, they often woke up and showed another side of themselves. As one student said of a stroll in the park:

When she was sitting in her own world, enjoying the sun, she came up with a comment I had never expected from an 80-year-old lady, and it was: “Yes, I am sitting here, looking at the water and the selection.” I asked, the selection?, “Yes, there are many handsome boys here.” This opened up our relationship, later I could ask, “Have you seen any handsome boys lately?” (Abigail)

Being in the real-world—for example, by sitting outside the nursing home—often changed the residents. When a group of residents was visiting the cinema together with some students, they enjoyed a snack and a soda, laughing at the right places in the movie. “One of the men exclaimed when he saw a woman with a nice-looking body, ‘Wow, look at that butt!’” (Veronica)

Getting hand-massages and manicures was a popular activity among the residents. Hand-massages were appreciated by both women and men. The residents expressed joy and satisfaction over getting hand-care and manicures. The touching of the hand and skin seems to touch the resident deeply. The satisfaction was expressed by smiling, laughing, and other expressions of joy. Several of the women were proud of their beautiful nails and showed them to the caregivers and other residents. One student gave a manicure to one of the residents even though one of the nurses said that the resident had never liked manicures or embellishing herself. In the reflection note, the student reported the following:

“The resident beamed like the sun and proudly showed her nails. She thanked me so many times for what I did for her. She cared for me as well and asked me how I was. When I stretched my back a couple of times, she asked me if my back was aching because I worked so thoroughly with her. She asked me several times if I was feeling well. Afterwards… I registered the resident’s non-verbal reactions: open eyes, smile, laughter, she was very aware if I was feeling well, proudly showed her hands while I was doing them and also when they were done. I also got a kiss on the cheek followed by laughter.”

(103)

The students often reported relaxation of the body muscles as an expression of joy, and many residents opened up and started to tell the students about their lives. Many of the residents were also so relaxed after the massage, the soft light, the music, and being read to that they fell asleep. The students also recognized that many of the residents were uncertain about what they remembered and could do, and therefore, needed support and affirmation:

“It seems that they have very low self-esteem. They sat down like this; no, I cannot play this game. When I said, come and sing along with us, they said, I do not know these songs. I said, now I sing, and you can hum if you know the melody. Suddenly, everyone could sing the songs, even though they had said they could not. They would not admit they could, in case they could not. If I said the title of a song, it was not useful. Because they usually do not recognize the title, but when you start singing, they will recognize the text.”

(Mia)

Many of the residents that were regarded as suffering from total loss of short-term memory, could recall episodes they had done together with the students: “four hours later, she came to me and said that it was so nice looking at the photos together today” (Emma). It appears that many of the residents gained mental clarity and began to remember when they came out into fresh air or were doing something that was meaningful to them. Many activities also seemed to bring forth memories:

“A popular activity was going to the city center and having coffee and cakes at a pastry shop. One of the female residents who enjoyed an old venerable patisserie remembered that as a little girl, she had been here with her mom. She was in a good mood for a long time. Usually, this resident used to be skeptical and suspicious.”

(Sophia)

One of the most important things for the residents was that someone was giving them time. It was not always the amount of time, but the fact that the resident felt someone was there for them. Having somebody sitting down with the residents and treating them as individual persons helped to improve the mental state of the residents. The residents showed expressions like “the face lights up”, and they started to talk, feeling that someone had the time to listen to them. It was important for residents that they could complete their stories without being interrupted.

## 4. Discussion

The aim of this article was to explore different ways to develop appropriate person-centered activities for nursing home residents based on what would be experienced as meaningful by them. We wanted to contribute to the discussion on increasing the quality of care in nursing homes, seeing the residents as persons and not only as a category of patients. When the residents were treated like individual persons and seen as real persons it meant that the establishment of a relationship had been successful and that such connection could enhance the continued work with the individual resident [12]. We found that the residents were enlivened by sensing the spark of life and that the students had to find ways to journey with the residents, while being guided by expressions of enlivening verbally and non-verbally. While most articles emphasize in more general terms the nurse-patient relationship and interaction [15,16], our findings provide detailed examples of how meaningful activities for nursing home residents can be achieved.

### 4.1. Journeying to Meaningful and Enlivening (Enjoyable) Activities

To assist the individual in finding and carrying out meaningful activities, the nurse/student needs to know the biography of the person. The nursing home residents used to enjoy different activities and encounters earlier in life and still do, even when living in the nursing home. This study focused mainly on person-centered care and individually tailored activities, assessing what provides the individual person with joy and the spark of life. Meaningful activities do not have to be extraordinary. Meaningful activities can simply be an awareness of including the patients in daily activities [40]. Of course, the activities must be person-centered and tailored to the individual person’s interests and resources. By using the residents’ resources, including the residents in daily activities can be a win-win situation. The caring staff can use daily activities to help the residents contribute their own resources instead of doing all the practical tasks for them. The traditional task-oriented culture in nursing homes, where the staff do everything for the residents, tends to make the residents dependent on the staff for everything. A person who is dependent or is perceived as being dependent on others for all care is very vulnerable in terms of maintaining their personal dignity, sense of self-worth, and self-respect [15,16]. According to Jacobson [41], the social process of dependency, and being forced to rely upon others for basic needs, violates a person’s dignity. Baillie et al. [42] also argued that a task-oriented culture can be a barrier to dignified care. In our study, as one of the students was giving a hand massage and a manicure to a resident, she became aware of and appreciated the empathy that the old woman showed her. It also helped her understanding the importance of tactility in caring for the resident. By accepting the friendly gesture from the resident, the student contributed to promoting the resident’s dignity of self, as described by Jacobsen [43].

Some activities presented in this article facilitated and contributed to more social interaction amongst the residents. For example, the students described that when they started singing or playing board games, they included several residents in the activity, eliciting laughter and engagement. Studies have shown that persons, especially those diagnosed with dementia, do not interact even if they are in the same room [8,44]. In this case, the students found that they functioned as social-action agents. It seems that in some group activities, the residents were stimulating each other but needed the nursing student as a catalyst for the interaction.

To understand how to assist persons with mental and physical impairment, the personnel need adequate and appropriate education [9]. Benner et al. [45] stated that we need a radical transformation of nursing education with a focus on formation of the person; which requires personal commitment and involves learning on multiple levels, including the intellectual, emotional, creative, imaginative, and physical (bodily sensations) levels. Likewise, Schwind et al. [46] argued that educational approaches for learning person-centered care are best fostered using creative, experimental, and reflective processes.

To raise the quality of care, it is necessary to have a holistic view of the residents. We will argue that it is not enough to focus on diagnosis and physical care. Care must also facilitate meaningful days with the opportunities to make personal choices, engage in social contact, and participate in meaningful activities. The findings show how a nurse can help the nursing home resident to discover meaningful and enlivening activities. To feel safe and confident in the journey, the resident needs the nurse to show interest, engagement, and spend time with them [16]. The students engaged actively with the residents, trying to find out what would enliven the person and provide a spark of life. The students chose to be participants rather than spectators in the residents’ lives.

### 4.2. How the Students Recognized and Interpreted the Expressions of Enlivening among the Residents

In our study, the students were encouraged to become involved with the individual residents, for whom they were responsible, to try to see them as persons, and to use their own intuition in “reading” the resident. By observing and interpreting what provided the spark of life and enlivened the individuals, they were doing more of the same and observing if the activity still resulted in the same enjoyment. On the other hand, the nurse/student must understand that, sometimes, residents refuse to do the things they used to like, which has to do with anxiety and insecurity. In that case, the helper must be there for the resident and to help the person overcome the resistance. Furthermore, the observations were documented both in the students’ logs and in the residents’ journals. By assessing the residents’ past and present interests, we consider, safeguard, and strengthen the residents’ resources. Edvardsson et al. [47] described promoting a continuation of the self and normality, knowing the person, and providing meaningful activities as some of the core variables for person-centered care. Our study shows that when the students were accompanying the residents outside of the institution, in “a normal life setting,” such as a restaurant, a cinema, or a park, the residents started to behave differently, and the students got to know them more as individual persons. These findings could indicate that striving for normality in long-term care settings could help residents to retain a continuation of the self. On the other hand, this also hints at the two-way mechanism: that the students upon entering through the door of the nursing home enter into an institution with a different set of normative understandings about the inhabitants, differing from what they would have had, if they had entered into the homes of the elderly.

Another interesting finding in the study shows that by doing meaningful activities that gave them the spark of live, the residents remembered the episode much longer than they usually did. This finding needs to be explored further. The residents exhibited several expressions of enlivening. The expressions interpreted as indicative of enlivening among the residents were both verbal and nonverbal. Even though there are different activities that seem to enliven different individual persons, there were some common features. The residents expressed enjoyment and became energized, especially in relation to being outside and sensing the wind and the sun on their skin. Most residents, like the rest of the Norwegian population, used to have a close relationship to nature [48], the four seasons of the year, and the changing weather, particularly enjoying the sun.

### 4.3. Limitations and Possibilities

This study builds on a mandatory assignment for first year students in nursing homes where they had to spend time with residents, search for meaningful activities to carry out together with them, and write reflective logs. Published research from the students’ process in this project [11] revealed that many of the students moved from an external and outward motivation to an internal motivation as they discovered how tailored meaningful activities could enliven the day for the residents. Students in this study received some critical comments related to the sustainability of their project when they had finished their placements. This could be interpreted as students bringing with them a different set of norms than were practiced in the nursing home, motivating them to see and understand the residents and the effort it takes to practice person-centered care in a different way.

The research reports on many obstacles for person-centered care in nursing homes. Lack of time and a culture of task-ordination rather than individualized care [18] together with a lack of leadership valuing the staff to be skilled and empathic in planning programs for the individual residents are some of the obstacles to person-centered care [12].

Leaders in a nursing home are key to changes in the nursing culture and to move towards individualized care including meaningful activities as a part of ordinary care. The joy of seeing the enlivenment and spark of life in the residents with a diversity of impairments can serve as a motivation, we believe, to continue with person-centered care. In addition, we believe it will also contribute to job satisfaction.

## 5. Conclusions

There are many ways to facilitate the enhancement of the quality of life for individuals in nursing homes. Our study shows that the student/nurse can be of great importance for the quality of care, especially when it comes to frail residents. Taking the time and effort to be led by the individual resident and tailoring activities that are meaningful, enhancing, and enabling for the individual person can enliven the person and bring joy to their life.

The challenges to finding meaningful activities for residents with poor communication skills can be overcome by carefully assessing their body language; as one student said: “the residents expressed emotions with their mouth and eyes, and their body posture, and they were more awake” (Birgit). Such reading of body language has been scarcely researched and discussed.

## 6. Clinical Implications

It is possible to find meaningful, enlivening, and person-tailored activities for persons with dementia and impaired cognitive function. By “journeying with” and engaging with the individual resident, the nurse/student can contribute to the resident feeling enlivened, waking up, and experiencing the activity as meaningful. This requires that the nurse/student be willing to “travel together” with the resident, using their creativity, sensitivity, and the try-and-fail approach in order to explore what is enhancing and enlivening for the individual person.

## Data Availability

Data used and analyzed in this study will be promptly available for the publisher upon request.
